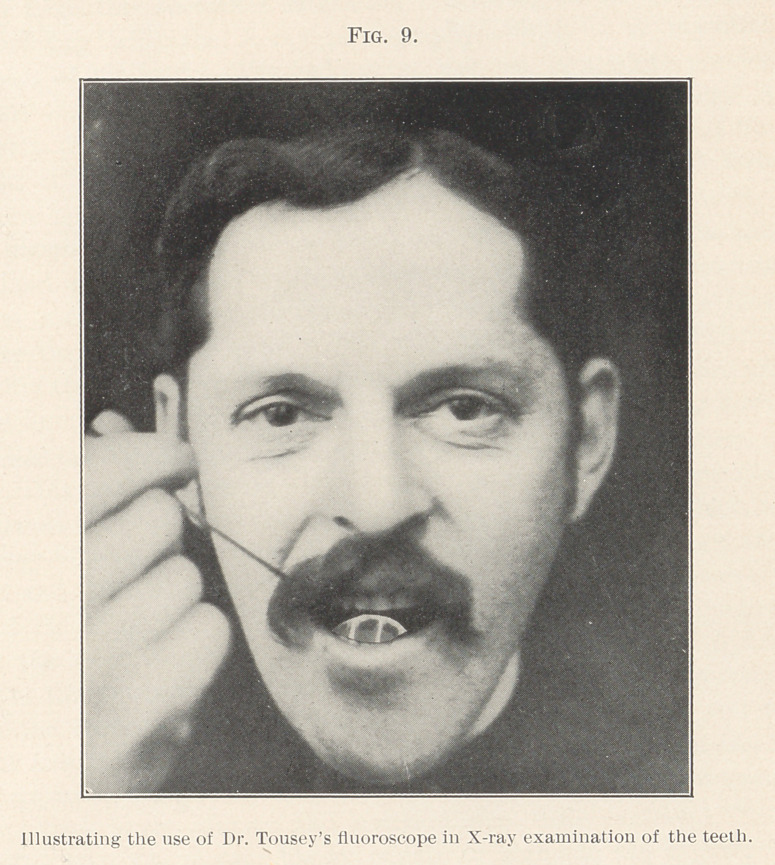# Radiotherapy in Pyorrhœa Alveolaris, and Dental Radiography1Read before The New York Institute of Stomatology, March 1, 1904.

**Published:** 1904-07

**Authors:** Sinclair Tousey


### RADIOTHERAPY IN PYORRHŒA ALVEOLARIS, AND DENTAL RADIOGRAPHY.[1]

### A DEMONSTRATION OF THE X-RAY, THE ULTRA-VIOLET RAY, HIGH-FREQUENCY CURRENTS, AND RADIUM, THE AUTHOR'S X-RAY TUBE FOR THE TREATMENT OF THE TEETH, AND HIS METHOD OF RADIOGRAPHY.

BY SINCLAIR TOUSEY, A.M., M.D.[2]

During the past two years a very great deal has been done with the X-ray and kindred applications as an adjunct to the mechanical and chemical treatment of the fatal disease of the teeth known as pyorrhœa alveolaris, or Riggs's Disease. During this time reports upon the subject of pyorrhœa have been published by Finsen, Custer, Parker, Hickey, Guy, Schwartz, Robin, Römer, Achorn, Suye, Talbot, Logan, Stewart, Newell, Goadby, Burchard,

[1] Read before The New York Institute of Stomatology, March 1, 1904.

[2] Attending Surgeon St. Bartholomew's Clinic. Assisted by Leo Green, D.D.S., of the Dental Department of St. Bartholomew's Clinic.

Ames, Grieves, Cook, Bödecker, Choteau, Rhein, Peacock, Bester, V. Wolrozynickie, and in *Dental Annals,* 1903, *Odontologie,* 1903, *Pathologie der Zähne,* 1903.

From the reports we gather that the probability is that the ordinary pus organisms, such as the staphylococcus, the bacillus pyocyaneus, bacillus coli communis, etc., have no direct share in the production of pyorrhœa alveolaris, and that the pneumococcus is also absent. Probably a member of the yeast family is the pathogenic germ. The constitutional conditions are often due to poisoning by toxins, and a filtered broth culture from these teeth kills guinea-pigs (Goadby). The constitutional cause is frequently rheumatism or gout (Newell). The teeth themselves are generally free from caries and the dental tissues hard and highly organized. Some cases (1) arise from a primary gingivitis, with the formation of hard, scaly, dark calculi beneath the gum margin. In other cases (2) the gingivitis is not marked, early deposits may be absent, and there is phagedenic pericementitis. In still other cases (3) degeneration and necrosis of the pericementum and deposits of calculi occur upon the lateral aspects of the tooth root, the gum margin being normal (Burchard).

The clinical appearance is described by Guy in an article in the *Dental Record,* reviewed in the *Dental Digest* for September, 1903. In his patient there was chronic enlargement of the submaxillary glands; the lower incisors, cuspids, and premolars were all very loose; the gum festoons hung patulous away from them; the gums were unhealthy, spongy, livid, and almost purpuric; pus exuded freely from about the roots of the teeth; the two upper incisors and a number of roots required extraction. The teeth were hypersensitive to heat and cold, and the patient had to warm his beer and cool his tea, and was quite unable to use his teeth.

In one of my own patients, the radiograph of whose lower front teeth, taken by the process described later, is shown herewith (Fig. 1), there was such great pain all along the right half of the lower alveolar margin as to make her sick in bed for several weeks, during which time the dentist had to visit her twice a day. She came to me six months later for constitutional treatment for indefinite digestive distress, with rheumatic or neuralgic pains, and with an excess of uric acid and a large amount of sugar in the urine. This is a condition which yields to the application of high-frequency currents and vibratory massage, and these were

Fig. 1.

Fig. 2.

Fig. 3.

applied over the abdomen and spine, and over the affected joints. A few applications of the X-Ray by my special tube were made to the teeth. The result is apparently a perfect cure, but could not have been accomplished without the local applications by the dentist.

Turning now to a demonstration of X-ray and phototherapeutic methods and apparatus, I call your attention to some X-ray pictures.

Fig. 2 is a radiograph of the chest and shoulders of a patient whom I am treating for tuberculosis of the larynx and lungs. The picture shows the right shoulder-joint and the shoulder-blade completely, with the ribs showing through the shoulder-blade, the picture being taken from behind. It also shows both clavicles. Shining through the entire thickness of the chest, it shows the heart area, and the area of the stomach, liver, and spleen. In three weeks' treatment the expectoration, which had been so profuse as to choke her, and had been full of the tubercle bacilli, entirely ceased; and at the end of a month's treatment the larynx was examined by a throat specialist, who found that the area of ulceration and redness below the vocal cords had decidedly diminished in size and that the hoarseness of the voice had much improved. She had also gained in weight and strength.

Fig. 3 is a picture of the hand and forearm which was taken at the patient's home, she being an old lady who had sustained an injury to the wrist which was feared might be a fracture. The picture was taken to make sure that the bones were in the correct position. It was taken right through the splints and bandages. It shows no fracture, but is interesting as a beautiful picture, and also because it shows rheumatic enlargement of the finger-joints.

Fig. 4 is the elbow-joint of a patient whom I was treating for chronic rheumatism by use of the X-ray and high-frequency currents, and who has spent a quarter of his time for the last twenty-seven years in hospitals being treated for rheumatism,—being filled with medicine, having his joints baked with a temperature of three to four hundred degrees, and being treated by different forms of electricity. A few months of treatment by means of the X-ray and high-frequency currents have made a new man of him. The picture shows the lower end of the humerus with the two condyles and the olecranon fossa, at which point the bone is transparent and must be very thin. It shows the radius with its head taking part in the formation of the elbow-joint and also articulating with the ulna, and shows the ulna overlapping the radius, the entire outline of both bones being visible. The olecranon process is clearly shown.

Fig. 5 is a picture of the teeth of a young lady in whom there is such a wide separation between the upper central and incisors as to present a disagreeable appearance and cause a certain degree of hissing in the voice, and the occasional flying out of a little drop of saliva in the face of any one to whom she might be talking. This is a patient of Dr. Leo Green, at whose request I took this picture in order to make sure that there was no supernumerary tooth or other reason why these teeth should not be brought together. The picture shows that there was nothing between the roots, and he accordingly regulated the teeth.

For the X-ray light we get the power by means of the street current which comes into the office as a continuous direct current of one hundred and ten volts. It has to pass through an interrupter, which consists of an outer jar in which one of the lead connections drops into dilute sulphuric acid, and an inner jar in which there is another lead connection also in dilute sulphuric acid. This inner jar has a certain number of perforations which permits of the passage of an electric current from the outer to the inner plate. As soon as the current is turned on hydrogen and oxygen gas are produced by the decomposition of this liquid by the current, and bubbles of this gas block up the small perforations in the inner jar, and in that way interrupt the current. No sooner is the current interrupted than the bubbles escape to the surface of the liquid and the current begins to flow again. This interruption takes place in some of my interrupters at the rate of ten thousand times a minute, and in others still more rapidly, and thus we have a series of ten thousand currents a minute and an intensity of one hundred and ten volts through the primary coil, each one producing a current of very much greater intensity in the secondary coil; the intensity in the second coil being dependent upon the very large number of turns of wire. In that coil there are about one hundred thousand feet of wire, so that when the current is turned on full we have about one million volts in the secondary coil.

This current is allowed to pass through the X-ray tube, which contains a partial vacuum through which the current is carried,

Fig. 4.

Fig. 5.

not as a tremendous spark or flame, as would be the case if the two points were in the open air, but as a very fierce bombardment of molecules which strike on the platinum disk in the centre of the tube and there break up into the form of motion which we know as the X-ray.

To see the bones of the hand, etc., by means of the X-ray, we have to employ a fluoroscope, which consists of a box into which we look, the bottom of it being coated with a chemical substance which becomes fluorescent under the influence of the X-ray. Very often people looking through the fluoroscope will be surprised when they discover later that the bottom of the box is an opaque piece of cardboard, the fluorescence being so brilliant that it seems as if they were looking through a piece of glass. Every substance is more or less transparent to the X-ray, but the more solid substances—metals and bones—cast deeper shadows than those less solid, like the flesh, etc. A piece of glass is relatively quite opaque to the X-ray, while wood is very transparent. Aluminum is very transparent, but lead is very much less so, and very often when treating a patient with the X-ray and in taking an X-ray picture we use a piece of lead to protect the portions which we do not want exposed to the rays.

The degree of penetration and the brilliancy of the light can be regulated by means of raising or lowering the degree of the vacuum in the tube, and all of the best tubes are provided with arrangements for this purpose. Of course, the strength of the current also has to be adjusted to the purpose in hand.

The ordinary X-ray tube, which I here show (Fig. 6), has its main portion spherical and the entire half of the tube in front of the plane of the platinum disk is brilliantly lighted up by a green light. The special X-ray tube which I have devised for the application of the X-ray to the treatment of Riggs's disease is made of lead glass, opaque to the X-ray except a cylindrical prolongation, from the end of which the rays go in a straight line, and none of the X-ray goes in any other direction, the light being absolutely localized to an area about an inch and a half in diameter, and the end of the tube is so shaped as to be convenient for application to the gums. If a great many treatments are necessary, of course the X-ray, if allowed to shine through the lips, would eventually cause a loss of the hair upon the lip, so that in such cases this special tube of mine ought to be applied with the lips separated.

For a few applications, or for the purpose of taking a picture, the X-ray can be allowed to shine right through the lips without any disturbance of any kind being produced.

The process by which I take pictures of the teeth, the roots of the teeth, supernumerary teeth, fractures of the jaw, etc., employs a piece of sensitized paper which is wrapped in opaque black paper and protected by thin rubber tissue; it is placed inside the mouth and pressed against the jaw, the light being allowed to shine from the outside of the face, and it is not necessary to use a special X-ray tube, the large spherical X-ray tube being perfectly adapted to the purpose. The distance is about ten inches, and the time of exposure required is from twenty seconds to a minute.

After making this exposure, we will take the piece of sensitized paper out of this envelope and drop it into a developing solution, and then later drop it into a fixing solution, and in five minutes we have a complete picture which may very probably show not only the roots of the teeth but also the entire pulp-cavity of the teeth extending down to the tips of the roots. In one picture, which I show you here (Fig. 7), we can see a gold crown upon the second molar, with a root filling from the same extending down about one-thirty-second of an inch into each root, instead of all the way; as it was supposed to be. The adjacent tooth, which appears to be sound, shows very perfectly the entire pulp-cavity of the root-canals. Pictures of this sort are, of course, necessary in making the diagnosis of supernumerary teeth, fractures of the jaw, impacted and displaced teeth, etc.

I now demonstrate to you the fluoroscope which I have devised for the immediate examination of the teeth and jaws with the X-ray. It is shaped like a dental mirror, but instead of a reflecting surface has a barium platino-cyanide surface protected from moisture by a sheet of transparent celluloid, and when in use instead of a reflection of the teeth we see a complete X-ray picture covering the whole surface of the fluoroscope. For satisfactory examinations the room ought to be darkened and the X-ray tube itself wrapped in a black cloth, these precautions being taken to exclude ordinary visible light and to cause the image upon the fluoroscope to become more brilliant by contrast. Figs. 8 and 9 show the appearance of this fluoroscope.

Another patient to whom I would like to refer is a lady whom

Fig. 6.

Fig. 7.

Fig. 8.

Dr. Tousey's fluoroscope for X-ray examination of the teeth and jaws.

I have treated for Riggs's disease by means of the X-ray and high-frequency currents. These high-frequency currents are produced by the same X-ray coil, with the addition of a D'Arsonal transformer and vacuum electrodes. These vacuum electrodes are simple glass tubes which contain a partial vacuum. They are of various shapes and are applied directly to the surface of the body, and when the current is turned on ten thousand waves of the violet-colored light pass down through the vacuum and disappear in the body every minute. The test by means of Willemite, which is also used for testing radium, shows the presence of ultra-violet rays in very rich abundance in this light. From these tubes there is produced a large amount of ozone right on the surface of the body, and this is carried in by the current. The electric current itself passes into a metallic handle which is held by the patient, and when the vacuum electrode is applied to the seat of disease the current passing through the patient has greater efficiency than can be applied in any other way.

The whole application is devoid of any uncomfortable sensation. In fact, there is practically no sensation except that of the actual contact of the glass with the surface. It is used by me with very great success in the treatment of rheumatism, gout, sciatica, paralysis, neuralgia, and as an adjunct in the treatment of tuberculosis.

One case of Riggs's disease which I have treated had been treated for a couple of months by Dr. Jones, a dentist in Birmingham, Ala. The treatment had been very successful, indeed, and consisted of almost daily applications of some caustic substance which destroyed the inflamed and necrotic tissue about the roots of the teeth. This subsequently gave place to a new and firm tissue, with the loss of only two teeth. She had been suffering from the disease for some six years before this course of treatment was undertaken, and when she came to New York there was very little of the original condition to be seen, and that little had disappeared entirely under the use of the vacuum electrodes and high-frequency currents applied through the lips. At the same time this patient has been cured of an epithelioma of the face by the X-ray.

The next part of the apparatus to be shown is the lamp which produces the ultra-violet ray. This is Dr. Piffard's modification of the Görl lamp, and is actuated by the X-ray coil with the addition of the Leyden jar, serving as a condenser. The lens in front of the lamp is made of quartz crystal, and, as you see by the experiment that I show you, the light produces a brilliant green fluorescence in a piece of Willemite; but this invisible ultra-violet ray will not pass through the thinnest piece of tissue-paper, and as I interpose such a piece of paper between the lamp and the Willemite you will see that the fluorescence is entirely prevented. The same takes place when a piece of glass is interposed between the lamp and the Willemite, although light appears the same when looking at it through a piece of glass.

The treatment of these conditions about the mouth, and also for lupus, consists in holding the lamp as close as possible to the affected surface. It seems to be something more than a merely antiseptic action which produces the benefit in these cases.

The next part of the apparatus to be seen is the Cooper-Hewitt light, a large cylinder of glass containing vapor of mercury, through which a current of very high intensity passes. The light appears white and is of four hundred candle-power, but the spectrum seems to be almost a pure violet, and anything red, like the beautiful bunch of roses which I have here, appears to be dark purple, from the absence of the red rays and the abundance of the violet element in the light. This light contains practically the chemical and life-giving properties of sunlight, about one hundred times intensified, and is applied directly over the bare chest at a distance of about four inches, and without danger of burning or discomfort. It is one of the parts of my treatment for tuberculosis.

The next part of the apparatus which I show you is the static machine, and its principal use for dental cases is for neuralgia; and I would very strongly advise any dentist, in treating a case of neuralgia apparently due to teeth, to do whatever is necessary in the way of treatment of the teeth, but not to consider extraction until the case has been treated by the static form of electricity. My own nurse suffered very much from neuralgia for two years, and two apparently healthy teeth were opened and the pulp-cavity disinfected and filled, but the neuralgia still persisted. The teeth were finally extracted, but the neuralgia continued worse than before, so that now for about a year she has suffered very much, indeed, in cold and wet weather, and has been unable to take solid food at such times. Finally, I gave her one or two treatments with high-frequency currents, which did not seem to agree with her; then about a month ago I gave her a single treatment with the static machine, which has resulted in the complete and entire

Fig. 9.

Illustrating the use of Dr. Tousey's fluoroscope in X-ray examination of the teeth.

disappearance of the neuralgia, although, as you know, there have been a great many cold and wet days since that time.

Now examine the specimen of radium, which, as you see, I keep in a small safe purchased for the purpose—the idea being not that I am so much afraid of losing it, as to prevent the radiation from this substance saturating everything in the room and interfering with the X-ray photography, which is so essential a part of my work. The radium, as you see, is a white powder about one-tenth gramme in amount, and its radial activity is twenty thousand times that of uranium. Now, if we turn out the light entirely, we see a faint glow, and holding a piece of Willemite near this tube, the Willemite is lighted up quite distinctly. A diamond, which I borrow from one of the gentlemen, is also lighted up. The other day a Spanish doctor from Porto Rico was here, and we exposed his diamond to these rays for such a long time that it became quite radio-active itself, and for the balance of the afternoon, instead of looking at the bones in his hand through the fluoroscope, I caught him every once in a while looking down at this sparkler on his finger, which was glowing in the darkness.

This electric vibrator which I show you now is a very essential part of the treatment for the uric acid condition upon which so many of these cases depend. It is applied over the abdomen and up and down the spine, and acts in a certain way like massage; but the vibrations are very, very rapid, and stimulate the action of the liver, intestines, stomach, and all spinal centres. I find it of the greatest service as an adjunct to high-frequency currents in the treatment of rheumatism, sciatica, and neuralgia, and in the treatment of obesity and of sluggish portal circulation, which come to so many people after middle life if they are prosperous and have heavy dinners and comparatively little exercise. Such ladies have the pleasurable experience of going to the expense of buying new corsets after taking in the old ones about two inches; and even gentlemen often take this treatment for the improvement in their figures and the very marked improvement in their condition of physical health.

Now we will take this X-ray picture, the patient being a young lady about twenty-three years old, with two temporary teeth (bicuspids) in the upper jaw and no evidence of permanent teeth, the question naturally being whether the permanent teeth are present and ready to make their appearance if these temporary teeth were to be extracted. The X-ray picture which we take is developed right here before you without requiring the use of a dark room, and shows no sign of permanent teeth. It shows that the roots of these permanent teeth are comparatively short, absorption having taken place very much as if the permanent teeth were present to take their place. Naturally, our advice to our friend, Dr. Green, is to preserve these primary teeth as long as possible.

Another patient shown now by Dr. Green is a woman about thirty-five, with pyorrhœa of the lower incisors, who shows marked improvement since the X-ray was begun at St. Bartholomew's clinic a few days ago. We will now treat this case with my special X-ray tube, applying the light directly to the affected gums for about three minutes, and this will be used twice a week.

### BIBLIOGRAPHY.

Harlan, A. W. Dental Hints, January, 1904 (reviewed from Items of Interest).
Parker, C. H. Dental Cosmos, December, 1903.
Ames, W. V. B. Dental Cosmos, May, 1903.
Grieves, C. J. Dental Cosmos, January, 1904.
Cook, Geo. W. Dental Digest, December, 1903.
Newell, E. B. Dental Digest, May, 1903.
Hickey, P. M. Dental Digest, May, 1903.
Guy, Wm. Dental Record (London), 1903, p. 162.
Bödecker, C. W. Dental Review, 1903, p. 110.
Schwartz. Montpel. Méd., 1903, p. 494.
Choteau. Dictionaire Dentaire.
Ames. Dental Cosmos, 1903, p. 355.
Rhein. Dental Cosmos, 1903, p. 369.
Robin. Journ. de Méd. de Paris, 1903, p. 135.
Custer. INTERNATIONAL DENTAL JOURNAL, 1903, p. 247.
Römer. Schweizer Vierteljahrschr. für Zahnheilk.
Achorn. Dental Cosmos, 1903, p. 189.
Peacock. Dental Record, 1903, p. 123.
Besten. Deutsche Monatschr. für Zahnheilk., 1902, p. 581.
Weil, C. Pathologie d. Zähne, 1903.
Odontologie, Paris, 1903, p. 365.
Schwartz. Archiv. d'Elec. Méd., 1903, p. 611.
Suye. Odontologie, Paris, 1903, p. 201.
V. Wolrozynickie. Wiener med. Wochenschr., 1903, p. 1245.
Talbot. Dental Summary, 1903, p. 538.
Logan. Brit. Dental Soc. Tr., 1903, p. 546.
Stewart. Memphis Med. Monthly, 1903, p. 345.
Dental Annual, 1903.

## Figures and Tables

**Fig. 1. f1:**
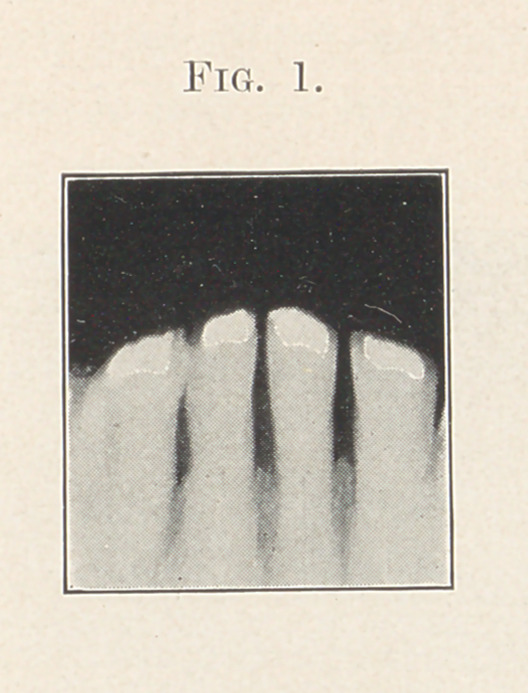


**Fig. 2. f2:**
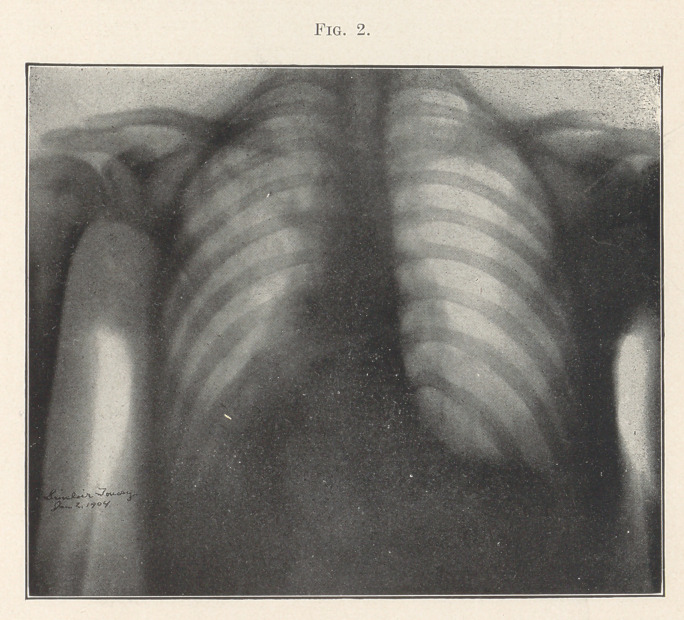


**Fig. 3. f3:**
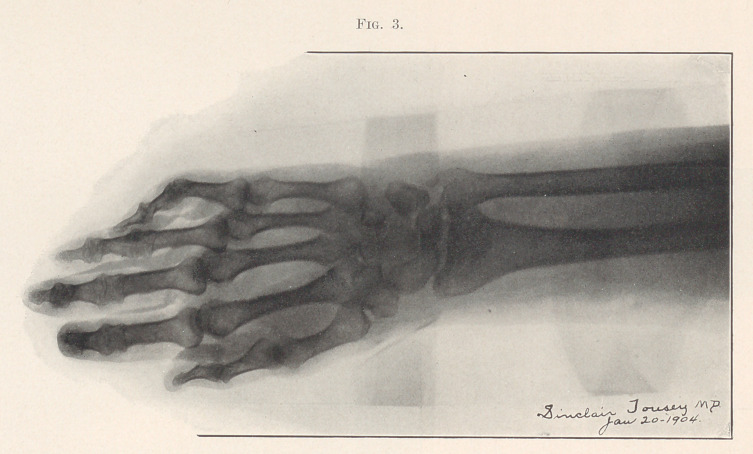


**Fig. 4. f4:**
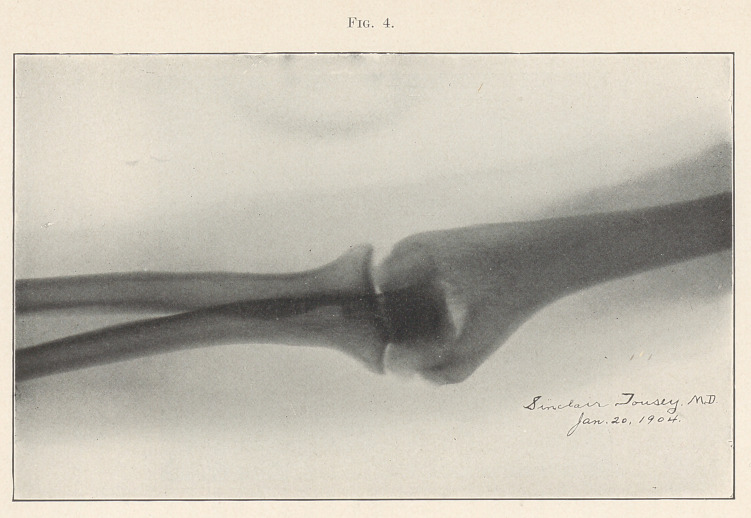


**Fig. 5. f5:**
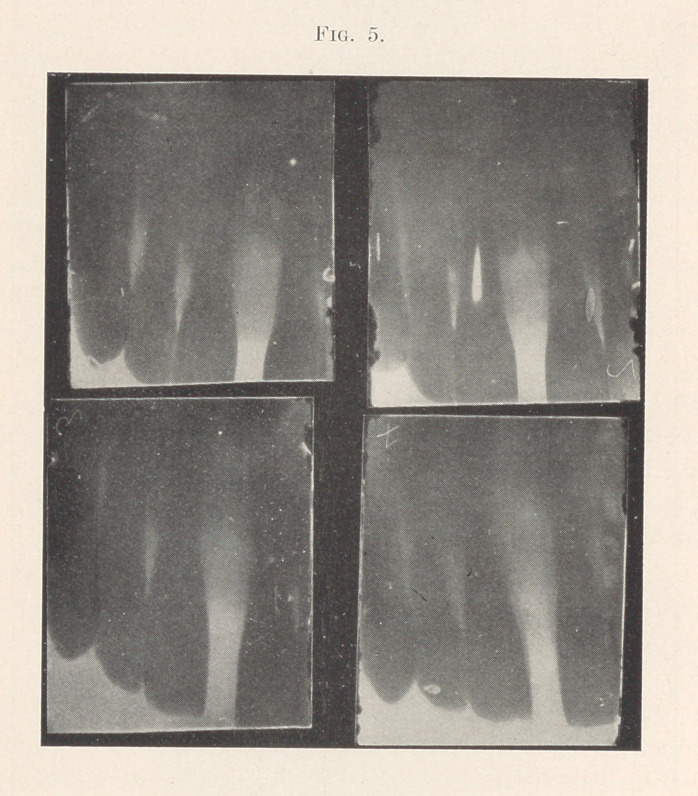


**Fig. 6. f6:**
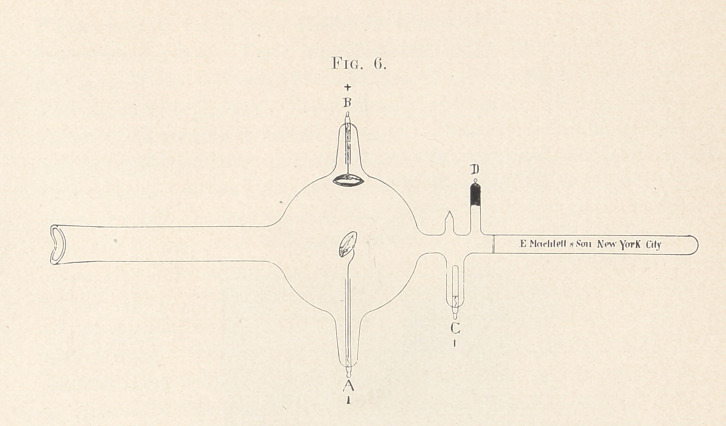


**Fig. 7. f7:**
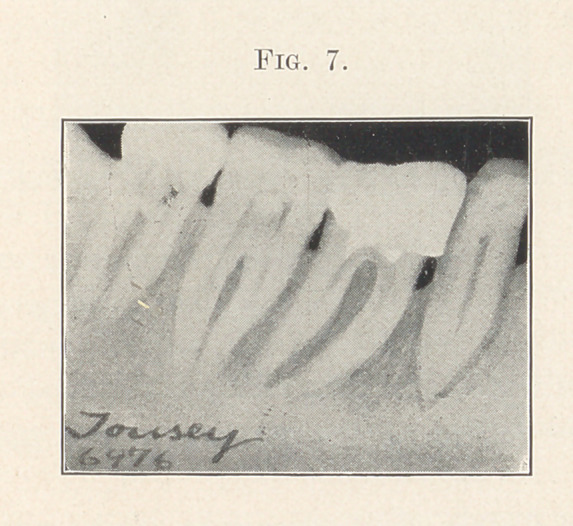


**Fig. 8. f8:**
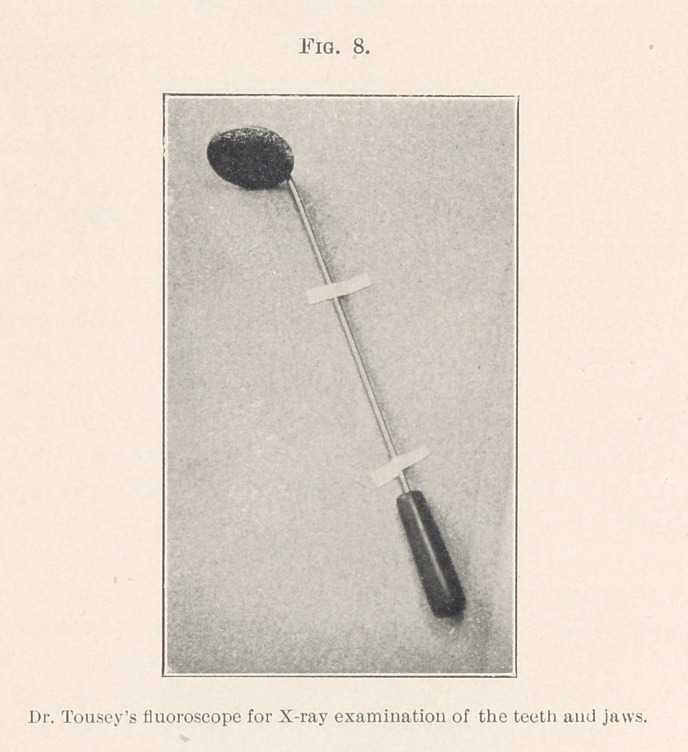


**Fig. 9. f9:**